# Construction of complex non-uniform rational B-spline volume parametric models with *G*^1^ continuity

**DOI:** 10.1186/s42492-026-00217-1

**Published:** 2026-04-09

**Authors:** Dan Wang, Long Chen, Jiahong Zhang

**Affiliations:** 1 School of Mechanical Engineering, University of Shanghai for Science and Technology, Shanghai, 200093 China; 2 School of Optical-Electrical and Computer Engineering, University of Shanghai for Science and Technology, Shanghai, 200093 China

**Keywords:** Volumetric parametric model, Creation method, Recreation method, *G*^1^ continuity

## Abstract

The construction of complex volumetric parametric models has long been a bottleneck in achieving integrated design and simulation modeling. To enhance model quality and simplify the modeling process, this paper proposes an innovative method for improving the continuity of complex volumetric parametric models. First, depending on whether the input consists of design parameters or surface models, a $G^0/C^0$-continuous volumetric parametric model is generated using either a creation or recreation approach. A new data structure is also developed to store the control-point indices and their topological relationships. Next, based on the actual connections among different patches in the volumetric models generated using the two aforementioned methods, the continuity conditions for three different scenarios are formulated. For each scenario, the corresponding systems of equations are established to determine the control points. Subsequently, an algorithm is developed to automatically sort, store, and adjust the relevant control points so that the volumetric parametric model satisfies the *G*^1^ continuity condition. The generated examples demonstrate that the volumetric parametric modeling method proposed in this study is effective for constructing complex models, significantly improving the model quality and rendering them more suitable for subsequent analysis and processing.

## Introduction

With the continued development of the manufacturing industry, the demand for computer aided design (CAD) and computer aided engineering (CAE) integration is steadily increasing. Unlike traditional finite element methods that require discretizing CAD models into mesh models, isogeometric analysis (IGA), proposed by Hughes et al. [1], enables direct simulation and analysis of spline-based models, significantly simplifying the product design process.

However, the current mainstream CAD systems use models in the boundary representation‌ (B-Rep) format, containing only boundary information and lacking internal details, rendering them unsuitable for direct use in IGA. By contrast, volumetric parametric models use spline-based curves and surfaces to represent both the internal and boundary features of the shape, rendering them directly applicable to IGA. When constructing volumetric parametric models, complex model structures must be divided into zero genus bivariate or trivariate tensor subdomains. These subdomains are then stitched together based on the principle that adjacent patches share common surfaces or curves, ultimately forming the complete model.

The methods for constructing volumetric parametric models can be categorized into two approaches: creation and recreation methods. The creation method typically involves model construction based on dimensions or sketches, whereas the recreation method facilitates the parametric reconstruction of the existing models, such as point clouds and mesh models.

When connecting different patches, continuity must be considered to better satisfy the design requirements and model quality standards. Continuity is measured in terms of parametric and geometric continuity. Following path connection, the shared boundaries of volumetric parametric models often achieve only $G^0/C^0$ continuity. However, for industrial products, $G^0/C^0$ continuity often fails to meet practical application requirements. Therefore, further improvements in continuity are necessary.

Extensive studies have been conducted on the continuity of parametric surfaces. However, research on parametric volume continuity is still in its infancy. Most methods for constructing continuous volumetric parameterized models focus on creating trivariate spline spaces [2–6] and the use of specialized splines to construct spline volumes [7–10]. However, limited research has addressed continuity adjustment when merging previously generated volumetric patches. Due to the three parametric directions in volumetric models, the connection process involves multiple boundary curves and surfaces that must satisfy several boundary conditions simultaneously. This renders the connection process more complex than for surface patch connection, highlighting the need for a systematic method to connect volumetric parametric blocks under the *G*^1^ continuity condition. A solid research foundation has been established for generating volumetric parametric models using both reconstruction [11] and creation methods [12, 13]. This study proposes a method for constructing complex volumetric parametric models with *G*^1^ continuity. The approach addresses control-point solutions across three connection types depending on the connection method. Examples are provided to demonstrate the effectiveness of the proposed approach. The key contributions of this study are as follows:

- A novel approach for constructing volumetric parametric models with enhanced continuity is proposed.
- An operation for merging complex volumetric parametric models while ensuring *G*^1^ continuity is presented.
- An algorithm for managing the data structure of complex volumetric parametric models with *G*^1^ continuity is developed.

The remainder of this paper is organized as follows. Related work subsection reviews the related studies. Methods for constructing volumetric parametric models subsection presents two volumetric parametric model construction methods. Connection of parametric volumes with *G*^1^ continuity subsection describes *G*^1^ connection of parametric volumes and explains the *G*^1^ continuity adjustment algorithm. Results and Discussion section provides specific application examples and discusses the advantages and applicability of this method. Finally, Conclusions section concludes the paper and outlines future work.

### Related work

Current research on volumetric parametric modeling primarily focuses on the following areas:

*Trivariate spline volume*. The construction of volumetric parametric models can be based on various types of spline functions, including traditional B-splines, NURBS, and specialized splines, such as T-splines, polynomial splines over hierarchical T-meshes (PHT-splines), and TH-splines. Wang and Qian [14] generated a trivariate tensor product B-spline solid based on six boundary surfaces, to ensure that the minimum Jacobian is positive. Massarwi and Elber [15] proposed a volumetric representation (V-rep) method based on trimmed trivariate B-splines. Massarwi et al. [16] proposed an untrimming algorithm to decompose trimmed trivariate B-spline volumes into a series of non-trimmed tensor product trivariate B-spline volumes using the subdivision method. Al Akhras et al. [17] proposed a method for constructing complex trivariate NURBS-based models with arbitrary topology, suitable for analysis using the standard B-rep models. Zhang et al. [7] generated genus-zero T-spline solids based on boundary T-spline surfaces. Wang et al. [8] proposed an algorithm for generating the trivariate T-spline solid of arbitrary genus from triangular mesh boundary models. Liu et al. [9] proposed a method for generating volumetric T-splines from an input B-rep model using solid geometry Boolean operations. Wei et al. [18] used truncated hierarchical tricubic splines (TH-spline3D) to construct volumetric parametric models suitable for adaptive isogeometric analyses. Chan et al. [10] proposed a method for constructing volumetric meshes suitable for the analysis based on PHT-Splines.

*Recreation method*. This method reconstructs volumetric parameterization models based on B-rep models. The primary targets are well-established B-rep models such as point cloud models, polygonal mesh models, finite element mesh models, and CAD standard formats such as the standard for the exchange of product model data (STEP). Methods such as polycube mapping and volumetric subdivision are used to generate the model’s internal structure, enabling the construction of a volumetric parameterization model. Li et al. [19] proposed the generalized polycubes method, which converts surface meshes into volumetric splines. Liu et al. [20] developed a method to generate rational solid T-splines by creating T-meshes from skeleton-based polycubes generated from input surface features. Chen et al. [11] developed a method for constructing volumetric parametric models of complex shapes based on simplified polycube structures. However, connecting volumetric blocks typically achieves only $C^0$ continuity.

*Creation method*. These methods include direct construction methods, Boolean operations, and interactive modeling through secondary development using commercial software. Peltier et al. [21] used a smooth skeleton generated based on a 3D stick figure to create a Bézier volume parametric model. Chen et al. [12] employed a segmentation, mapping, and merging mechanism to generate volumetric parameterization models suitable for IGA analysis. Although $C^1$ continuity conditions were provided for merging the blocks, no specific implementation method was presented.

Volumetric parametric models with only $G^0/C^0$ continuity often fail to satisfy the requirements for practical applications. Therefore, improving the continuity of these models is necessary. Relatively little research has been conducted on volume continuity. A review of studies on surface continuity is presented here. Collin et al. [22] investigated analysis-suitable *G*^1^ (AS *G*^1^) geometry parametrizations for achieving optimal approximation in $C^1$ isogeometric spaces over planar B-spline geometries and identified the phenomenon of $C^1$ locking. Kapl et al. [23] built upon Collin et al.’s [22] research to propose a method for constructing AS *G*^1^ planar multi-patch parameterizations. Oh et al. [24] established the necessary conditions for *G*^1^ interpolation at a singular vertex formed by intersection of the three boundary curves using Bézier surfaces. In cases where these conditions are not satisfied, they proposed a subdivision method that utilizes three rectangular subpatches, including T junctions to achieve *G*^1^ continuity. This study extends the method to achieve a *G*^1^ continuous connection between surface patches to *G*^1^ continuous connections of complex volumetric parametric patches.

Some studies approach volumetric continuity by constructing parametric spline spaces and basis functions. Chan et al. [4] achieved $C^1$ continuity between 2D and 3D multi-patch domains by constructing $C^1$ continuous basis functions through a linear combination of $C^0$ continuous basis functions over the multipatch domains. Birner et al. [2, 3] employed a gluing data method to generate a $C^1$ continuity isogeometric spline space over two trivariate patch domains and developed a theoretical framework to prove this numerical method. Kapl and Vitrih [6] proposed a method for constructing $C^1$ isogeometric spline function spaces and their associated basis functions over trilinearly parameterized multipatch volumes. Certain properties of the resulting $C^1$-spline space were examined by focusing on the construction of a specific class of multipatch volumes with a single inner edge. These studies largely emphasized theoretical aspects, and comparatively little research has been conducted on the practical construction of continuous connections in complex volumetric parametric models.

Xu et al. [25] applied energy optimization to single volumetric parametric blocks enclosed by B-spline surfaces, and generated multiple B-spline parametric volumes satisfying the $C^1$ continuity conditions. Burkhart et al. [26] performed isogeometric finite element analysis (IGA) on Catmull-Clark subdivision solids, achieving seamless integration between CAD and CAE. In addition, the Catmull-Clark elements maintain $C^2$ continuity at the boundaries and interior, except for the irregular vertices and edges. Qin et al. [27] created generalized Bézier (GB) polyhedra volumes based on GB surfaces, achieving *G*^1^ or $G^2$ continuity among volumes. However, their approach only considers connections along a single parametric direction and does not address complex connections across multiple directions.

The existing methods for constructing continuous volumetric parametric models use special splines or construct parametric spline spaces and basis functions typically based on reconstruction approaches, where the volume models are directly generated from the input surface meshes. These methods tend to emphasize the theoretical aspects. By contrast, interactive modeling approaches that create volumetric parametric models from the design parameters align more closely with practical design workflows. The proposed continuity enhancement algorithm can be applied to improve the continuity of volume parametric models generated by both reconstruction and creation approaches. It not only supports simple one-directional connections, but also addresses complex configurations such as multidirectional planar connections and 3D composite connections, thereby offering greater practicality in real-world applications.

## Methods

### Methods for constructing volumetric parametric models

**Fig. 1:**
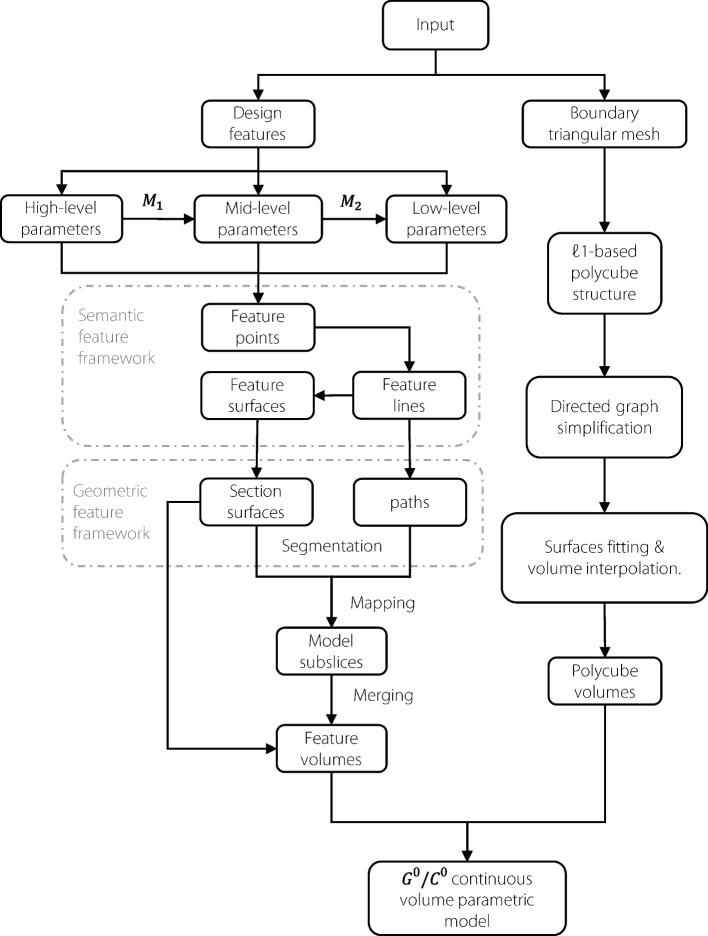
Generation process of the volumetric parametric model

The methods for constructing volumetric parametric models can be categorized into creation and recreation methods. An integrated design software for modeling, simulation and optimization (iMSO) of volumetric parametric modeling has been developed. The process of generating volumetric parametric models is illustrated in Fig. 1. The creation method constructs volumetric parametric models based on a segmentation-mapping-merge mechanism, whereas the recreation method relies on the directed graph simplification of $\ell _1$ polycube structure techniques.

#### Segmentation, mapping, and merging mechanism

The feature framework of iMSO is primarily divided into semantic and geometric feature frameworks. The primary purpose of constructing a semantic feature framework is to parameterize the features, enabling the layered control of the model parameters across three levels: high, medium, and low.

High-level parameters primarily control the positional dimensions of the model features, such as the position of the center of the hole relative to a rectangular block. Mid-level parameters define the shaping dimensions of the model features, such as the length, width, and height of the block, or the radius and height of the hole. Low-level parameters describe finer model details and control-point parameters, such as the positions of the control points when subdividing the circular hole. These three parameter levels form the foundation of the model, from which the feature points, lines, and surfaces can be derived.

**Fig. 2:**
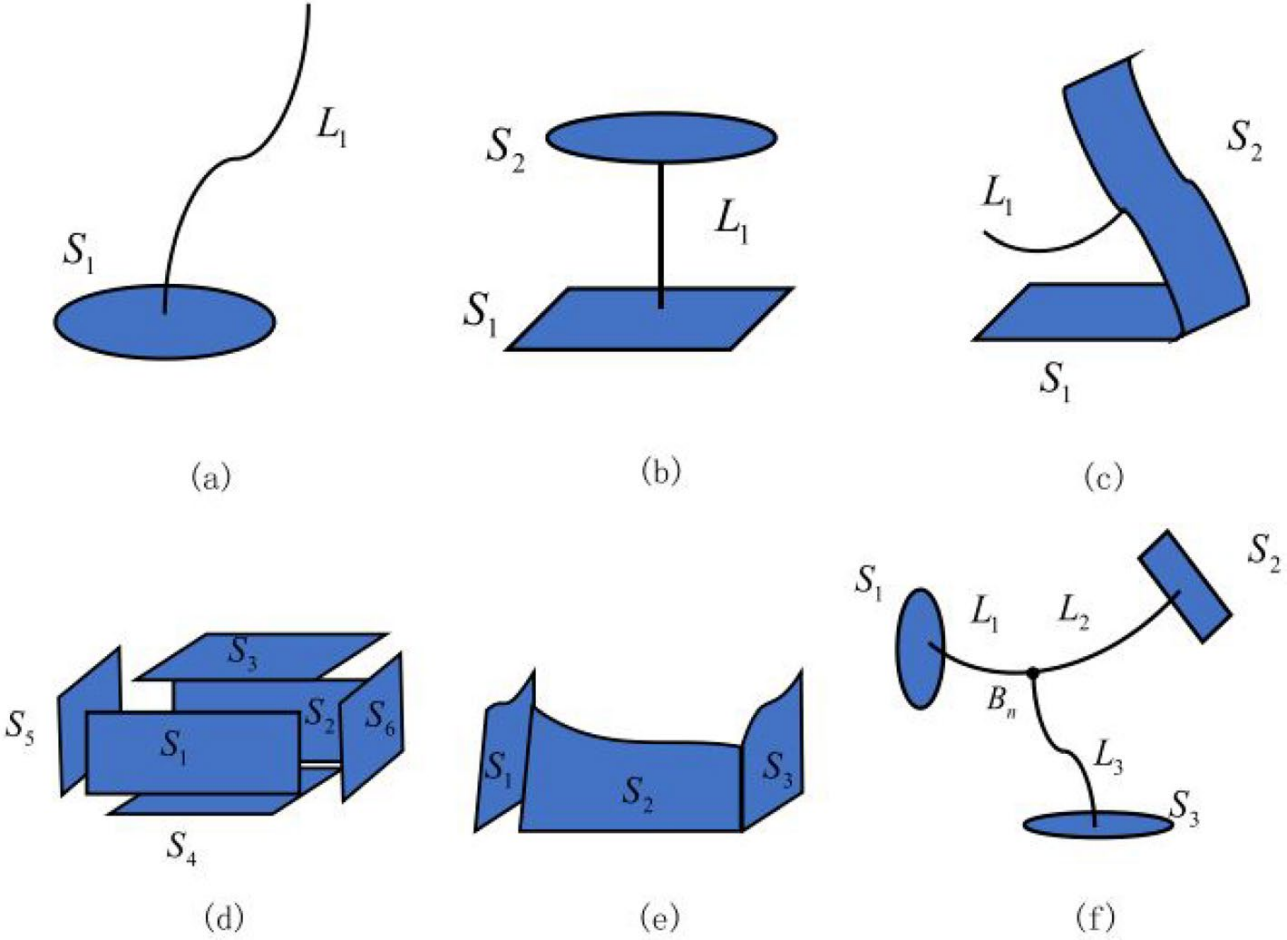
Types of geometric feature frameworks [13]

However, the resulting curves and surfaces cannot be used for direct modeling. The elements required for volumetric parametric modeling have to be extracted from them to construct the geometric feature framework. Based on the generation method of the volumetric parametric models (Fig. 2), the geometric feature framework can be divided into the following four types. The simplest type comprises a single cross section and a single path without any nodes. This corresponds to an extrusion semantic feature framework, as in common operations such as extrusion, rotation, and sweeping. The next type comprises multiple cross sections and a single path without any nodes. This corresponds to a lofting semantic feature framework that primarily involves lofting operations with multiple cross sections. The subsequent type comprises multiple cross sections without paths or nodes. This aligns with the second case in the interpolation of the semantic feature framework. It involves first assessing whether the cross sections need to be interpolated, and then using interpolation to construct the entire model. The final type comprises multiple cross sections and paths, with at least one node present. This corresponds to interpolation of the semantic feature framework.

NURBS volumes cannot be used to independently construct models at connection points; hence, the paths have to be split at the nodes first. Then, the nodes are handled separately before stretching the cross sections along the corresponding paths to construct the volumetric parametric model. These four types of geometric feature frameworks are the fundamental geometric feature framework types in iMSO. They can be combined to generate complex geometric feature frameworks.

Once the geometric feature framework of the model is constructed, it can be segmented as needed. The segmentation of volumetric parametric models includes two parts: feature segmentation (volumetric segmentation) and 2D geometric domain segmentation (quadrangular segmentation). Feature segmentation, or volumetric segmentation, is a decomposition step performed before model construction. It involves dividing the entire model volume into several feature bodies (also referred to as features, as indicated in the feature framework). These feature bodies must satisfy one of the following conditions: (1) Conform to one of the four previously described geometric framework features. (2) Generate by interpolating six convex quadrilateral surfaces.

The volumetric parametric models must be topologically hexahedral. The feature bodies obtained from the feature segmentation do not necessarily satisfy this requirement. Therefore, full quadrilateral segmentation is applied to the cross sections corresponding to the feature volumes. By executing the quadrilateral segmentation algorithm a set of boundary lines is obtained for all the quadrilateral subdomains, which are then used for interpolation to generate NURBS surface patches corresponding to the quadrilateral segments.

After these two segmentation operations are completed, appropriate modeling generation methods are selected based on the geometric feature framework corresponding to each feature volume. These methods include extrusion, rotation, sweeping, lofting and interpolation, to perform volumetric parametric mapping and obtain individual feature volumes. Finally, the feature volumes are connected together along their common boundary surfaces to obtain a complete volumetric parametric model.

#### Simplification of the $\ell _1$ polycube structure technique

The reconstruction method for generating volumetric parametric models in the iMSO platform is based on simplifying the $\ell _1$ polycube structure technique. This method can be summarized in the following three steps:

1. *Generation of an* $\ell _1$*-based polycube structure from an input boundary triangular mesh model.* The input triangular mesh is transformed into a polycube by minimizing the $\ell _1$-norm of the mesh’s normals.
2. *Simplification of the polycube structure using a directed graph approach.* The number of blocks in the generated model is minimized by using a directed graph approach to merge the flat patches.
3. *Surfaces fitting and volume interpolation.* Spline fitting is applied to the obtained polycube structure to generate boundary surfaces, followed by trivariate Coons interpolation of the boundary surfaces to create volumetric elements.

#### Visualization and evaluation of the constructed volume parametric model

**Fig. 3:**
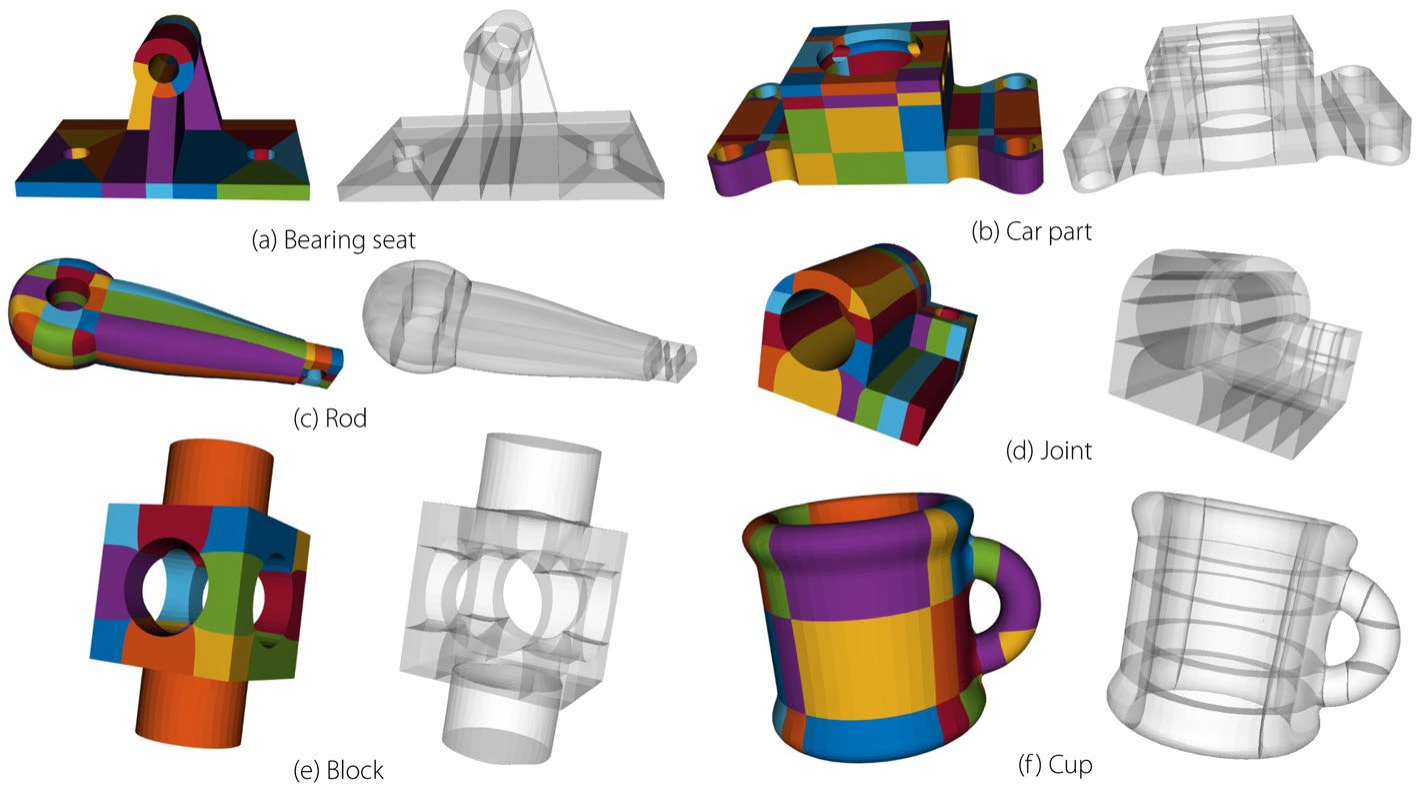
Volumetric parametric models generated by the segmentation, mapping, merging mechanism and polycube structure method

Figure 3 shows the volumetric parametric models generated using the segmentation, mapping, and merging mechanisms and polycube structure method, respectively. Volume parametric models with $C^0/G^0$ continuity often fail to satisfy the requirements for analysis and practical applications. Continuity has to be improved to enhance the quality of the volumetric parametric model.

### Connection of parametric volumes with *G*^1^ continuity

#### *G*^1^ continuity condition of simple face-to-face connections

**Fig. 4:**
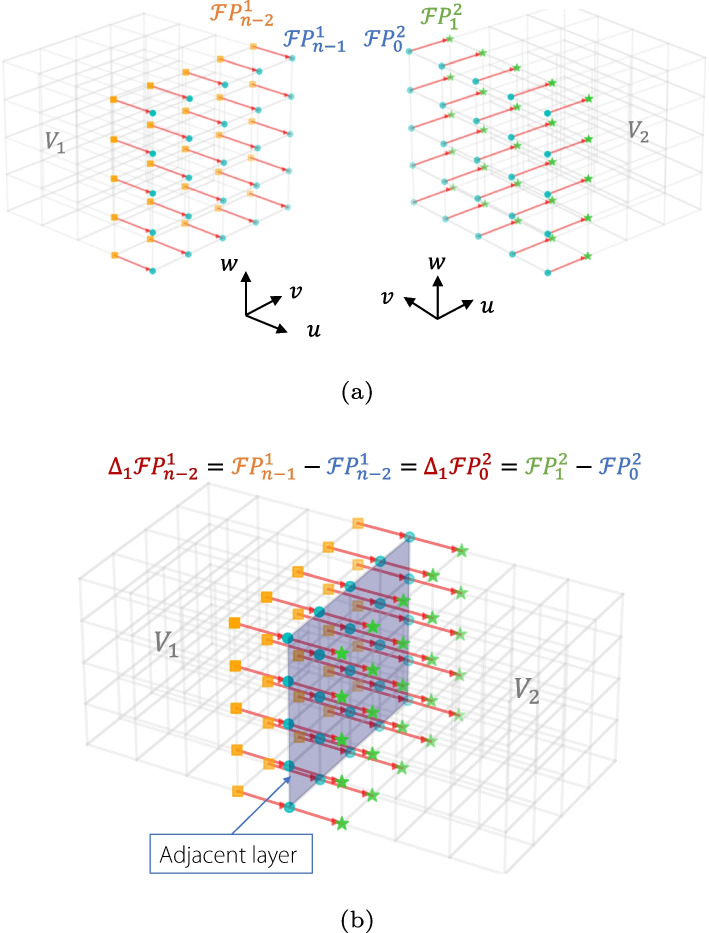
Inner control points related to the connection of two parametric volumes

When two volumes are connected along a common surface, achieving *G*^1^ continuity requires that the internal control points meet certain conditions. Consider $V_1$ and $V_2$ as two parametric volumes with a common boundary surface *S* (Fig. 4). The condition for achieving $G^0$ continuity at the common boundary surface is $V_1(1,v,w)=V_2(0,v,w)$. Conversely, the condition for achieving *G*^1^ continuity is [4]

1 $$\begin{aligned} \textrm{det}(\frac{\partial V_1(1,v,w)}{\partial u}, \frac{\partial V_1(1,v,w)}{\partial v}, \frac{\partial V_1(1,v,w)}{\partial w}, \frac{\partial V_2(0,v,w)}{\partial u})=0 \end{aligned}$$

Given that $V_1$ and $V_2$ share the same partial derivatives with respect to *v* and *w* at the stitching surface *S*, $\frac{\partial V_2(0,v,w)}{\partial v}$ and $\frac{\partial V_2(0,v,w)}{\partial w}$ can be disregarded.

Based on the properties of NURBS volumes, the *u*-direction $C^1$ and *G*^1^ continuity conditions are simplified as follows [27]:

$$\begin{aligned} C^1: \quad \qquad \Delta _1 \mathcal {F} \boldsymbol{P} _{n-2}^1= & \Delta _1 \mathcal {F} \boldsymbol{P} ^2 _{0} \\ G^1: \quad \qquad \Delta _1 \mathcal {F} \boldsymbol{P} _{n-2}^1= & \alpha \Delta _1 \mathcal {F} \boldsymbol{P} ^2_{0} \end{aligned}$$

where $\mathcal {F} \boldsymbol{P} ^m_i$ represents the *i*th layer control network of the *m*th volume. $\Delta$ denotes the iterative forward difference operator.

$$\begin{aligned} \Delta _0 \mathcal {F} \boldsymbol{P} _0= & \mathcal {F} \boldsymbol{P} _0^1\\ \Delta _1 \mathcal {F} \boldsymbol{P} _0= & \Delta _0 \mathcal {F} \boldsymbol{P} _1 - \Delta _0 \mathcal {F} \boldsymbol{P} _0 \end{aligned}$$

Therefore, the two rows of control points must be adjusted near the boundary surface, as shown in Fig. 4, ensuring that their connecting lines with the boundary control points are collinear with opposite lines to achieve *G*^1^ continuity.

However, because of the three parametric directions in the volumetric models, a wider variety of scenarios can arise during the connection of blocks than during the connection of surface patches. In addition to unidirectional face-to-face connections, there are also complex connection methods involving planar multipatches and 3D combination connections. The following discussion focuses on the continuity conditions of these two connection types.

#### *G*^1^ continuity conditions of complex connections

##### Planar multi-patches connection

**Fig. 5:**
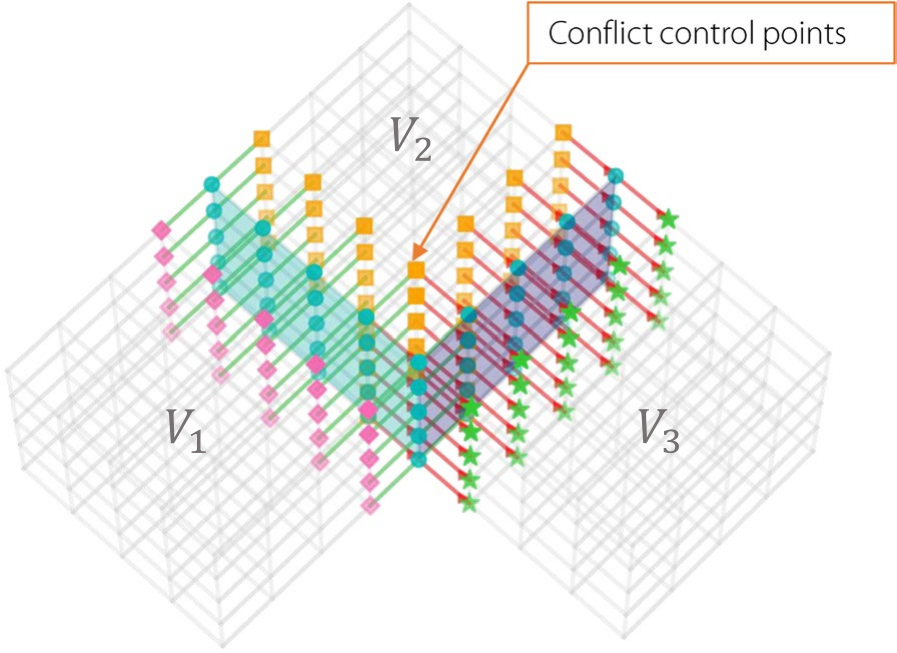
Conflict control points of three volume patches

Unlike the simple case of two-volume blocks, where only the continuity of control points along a single direction at the boundary surface must be considered, multiple blocks sharing a boundary spline (Fig. 5) involve connections along boundary lines with other blocks on different directional boundary surfaces. This can lead to conflicting continuity conditions at the internal control points. Therefore, it is necessary to solve for the internal control points that simultaneously satisfy the continuity conditions in both directions.

A row of control points inside the volume is located at the intersection of the lines connecting the control points for the continuity conditions on both boundary surfaces (Fig. 5). If the orange control points are adjusted based solely on the continuity conditions of boundary surface 1, they will inevitably affect the continuity conditions on boundary surface 2. The inner control points located at the intersection of the lines connecting the boundary control points for the continuity conditions are referred to as conflict points.

**Fig. 6:**
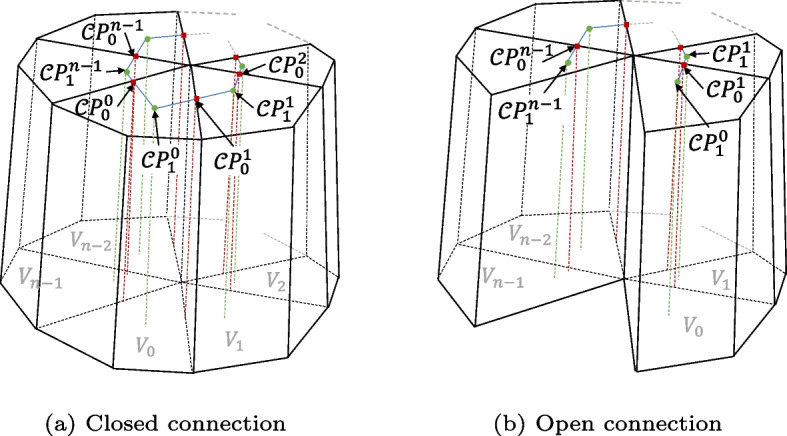
Two-directional connection of common boundary surfaces and splines

To obtain control points that simultaneously satisfy the continuity conditions in both directions, it is necessary to establish and solve a system of equations based on the continuity conditions of multiple boundaries. As shown in Fig. 6, depending on whether the starting and ending blocks are face-to-face, the arrangement can be categorized as open or closed.

Consider the case when *n*-volume patches are connected in a closed loop along a common boundary spline, with 2*n* columns of control points related to the boundary conditions ranging from $\mathcal {C}\boldsymbol{P}^0_0$ to $\mathcal {C}\boldsymbol{P} ^{n-1}_1$, where $\mathcal {C}\boldsymbol{P}^m_j$ represents the *j*th column of $\mathcal {F}\boldsymbol{P}^m_i$. The continuity condition system of equations for this scenario is

2 $$\begin{aligned} \left\{ \begin{array}{ll} \mathcal {C}\boldsymbol{P}_0^0-\mathcal {C}\boldsymbol{P}_1^{n-1}& =\lambda _1(\mathcal {C}\boldsymbol{P}_1^0-\mathcal {C}\boldsymbol{P}_0^0)\\ \mathcal {C}\boldsymbol{P}_0^1-\mathcal {C}\boldsymbol{P}_1^0& =\lambda _2(\mathcal {C}\boldsymbol{P}_1^1-\mathcal {C}\boldsymbol{P}_0^1)\\ & \vdots \\ \mathcal {C}\boldsymbol{P}_0^{n-2}-\mathcal {C}\boldsymbol{P}_1^{n-3}& =\lambda _{n-1}(\mathcal {C}\boldsymbol{P}_1^{n-2}-\mathcal {C}\boldsymbol{P}_0^{n-2})\\ \mathcal {C}\boldsymbol{P}_0^{n-1}-\mathcal {C}\boldsymbol{P}_1^{n-2}& =\lambda _{n}(\mathcal {C}\boldsymbol{P}_1^{n-1}-\mathcal {C}\boldsymbol{P}_0^{n-1}) \end{array}\right. \end{aligned}$$

This can be expressed in matrix form as follows:

3 $$\begin{aligned} \left\{ \begin{array}{ccccccc} 1& \lambda _1& 0& 0& 0& \cdots & 0\\ 0& 1& \lambda _2& 0& 0& \cdots & 0\\ \vdots & \vdots & \vdots & \vdots & \vdots & \ddots & \vdots \\ 0& 0& 0& 0& \cdots & 1& \lambda _{n-1}\\ \lambda _n& 0& 0& 0& 0& \cdots & 1 \end{array}\right] \left[ \begin{array}{c} \mathcal {C}\boldsymbol{P}_1^{n-1}\\ \mathcal {C}\boldsymbol{P}_0^1\\ \vdots \\ \mathcal {C}\boldsymbol{P}_1^{n-3}\\ \mathcal {C}\boldsymbol{P}_1^{n-2} \end{array}\right] = \left[ \begin{array}{c} (1+\lambda _1)\mathcal {C}\boldsymbol{P}_0^0\\ (1+\lambda _2)\mathcal {C}\boldsymbol{P}_1^1\\ \vdots \\ (1+\lambda _{n-1})\mathcal {C}\boldsymbol{P}_0^{n-2}\\ (1+\lambda _{n})\mathcal {C}\boldsymbol{P}_0^{n-1} \end{array}\right] \end{aligned}$$

When *n* is odd, the common boundary line becomes a singular line, and the boundary control points on this line are singular points. The coefficient matrix is of full rank, which means that the matrix has a unique solution:

4 $$\begin{aligned} \mathcal {C}\boldsymbol{P}_1^{n-2}=\frac{(1+\lambda _n)\mathcal {C}\boldsymbol{P}_0^{n-1}-\lambda _n(1+\lambda _1)\mathcal {C}\boldsymbol{P}_0^0+\cdots + \lambda _n\lambda _1\cdots \lambda _{2n-2}(1+\lambda _{n-1})\mathcal {C}\boldsymbol{P}_0^{n-2}}{\lambda _1\lambda _2\cdots \lambda _n+1} \end{aligned}$$

When *n* is even, the coefficient matrix is not of full rank and there are infinitely many solutions. Then the solution can be obtained through optimization, and the continuity equations are treated as constraints. Simultaneously, to preserve model geometry the objective is to minimize changes in the control-point positions. By iteratively solving for the optimal control-point positions, *G*^1^ continuity is achieved.

When *n* volume patches are non-closed and connected along a common boundary spline, the control points related to the boundary conditions range from $\mathcal {C}\boldsymbol{P}_1^0$ to $\mathcal {C}\boldsymbol{P}_1^{n-1}$, for $2n-1$ columns of control points. The equation set can be represented as

5 $$\begin{aligned} \left\{ \begin{array}{ll} \mathcal {C}\boldsymbol{P}_0^1-\mathcal {C}\boldsymbol{P}_1^0& =\lambda _1(\mathcal {C}\boldsymbol{P}_1^1-\mathcal {C}\boldsymbol{P}_0^1)\\ \mathcal {C}\boldsymbol{P}_0^2-\mathcal {C}\boldsymbol{P}_1^1& =\lambda _2(\mathcal {C}\boldsymbol{P}_1^2-\mathcal {C}\boldsymbol{P}_0^2)\\ & \vdots \\ \mathcal {C}\boldsymbol{P}_0^{n-2}-\mathcal {C}\boldsymbol{P}_1^{n-3}& =\lambda _{n-2}(\mathcal {C}\boldsymbol{P}_1^{n-2}-\mathcal {C}\boldsymbol{P}_0^{n-2})\\ \mathcal {C}\boldsymbol{P}_0^{n-1}-\mathcal {C}\boldsymbol{P}_1^{n-2}& =\lambda _{n-1}(\mathcal {C}\boldsymbol{P}_1^{n-1}-\mathcal {C}\boldsymbol{P}_0^{n-1}) \end{array}\right. \end{aligned}$$

The matrix form is as follows:

6 $$\begin{aligned} \left[ \begin{array}{ccccccc} \lambda _1& 0& 1& 0& 0& \cdots & 0\\ 0& 0& \lambda _2& 0& 1& \cdots & 0\\ \vdots & \vdots & \vdots & \vdots & \vdots & \ddots & \vdots \\ 0& 0& 0& 0& 0& \cdots & \lambda _{n-1} \end{array}\right] \left[ \begin{array}{c} \mathcal {C}\boldsymbol{P}_1^0\\ \mathcal {C}\boldsymbol{P}_1^1\\ \vdots \\ \mathcal {C}\boldsymbol{P}_0^{n-1} \end{array}\right] = \left[ \begin{array}{c} (1+\lambda _1)\mathcal {C}\boldsymbol{P}_0^1\\ (1+\lambda _2)\mathcal {C}\boldsymbol{P}_0^2\\ \vdots \\ (1+\lambda _{n-1})\mathcal {C}\boldsymbol{P}_1^{n-1} \end{array}\right] \end{aligned}$$

##### 3D composite connections

In the volumetric parametric models, each unit volume patch has three parametric directions: *u*, *v*, and *w*. A unit volume patch can connect with other patches along all three directions; hence, continuity conditions near corner connection points in three dimensions must be carefully considered. This subsection discusses the *G*^1^ continuity conditions in these cases where multiple unit volume patches are joined at the same corner point along three directions.

**Fig. 7:**
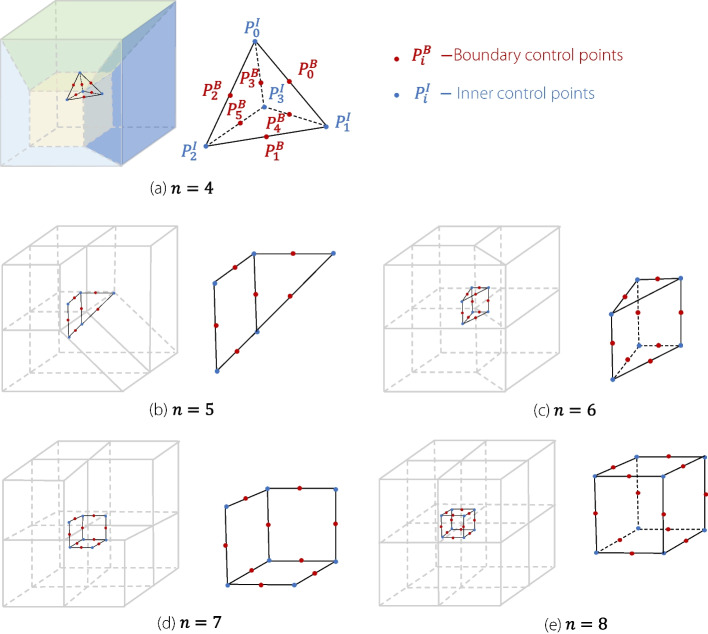
Multiple unit volume patches joined at one corner point

Figure 7 shows $4\sim 8$-unit-volume patches joined at the same corner point. When four volumetric patches meet at a corner point, there are four common boundary lines and four common boundary surfaces converging at the corners. Near the corner point, the control-point lines related to the *G*^1^ continuity condition form a triangular pyramid. The control points along each edge of the pyramid must be collinear to satisfy the *G*^1^ continuity condition. The vertices of the triangular pyramid are the conflict points. The points along the edges represent boundary control points. This type of connection can be viewed as a combination of three closed planar connections, as described in Planar multi-patches connection subsection.

Similarly, a five-volume patch connection can be considered as a combination of three-volume patches with open and closed planar connections, using four-volume patches with closed planar connections. A six-volume patch connection can be considered as a combination of two three-volume patches with closed planar connections and three face-to-face connections. A seven-volume patch connection can be considered as a combination of a three-volume patch open connection and three four volume patches with closed planar connections. Finally, an eight-volume patch connection can be considered as a combination of six four-volume patches with closed planar connections.

The conflict point must satisfy multiple continuity conditions. Therefore, a system of equations must be established and solved. The continuity conditions for the conflict points are as follows.

7 $$\begin{aligned} \left\{ \begin{array}{ll} (P_0^B-P_0^I)& =\lambda _0(P_1^I-P_0^B)\\ (P_1^B-P_1^I)& =\lambda _1(P_2^I-P_1^B)\\ & \vdots \\ (P_i^B-P_j^I)& =\lambda _n(P_k^I-P_l^B) \end{array}\right. \end{aligned}$$

$P_i^I$ are internal control points that must be solved. $P_i^B$ are the known boundary control points. The number of equations depends on the number of edges in the wireframe formed by connecting conflict points in various types of connections (Fig. 7). The 3D composite connections involve complex configurations that cannot be solved analytically, and therefore, requires optimization to achieve *G*^1^ continuity.

#### Searching method for boundary condition related control points

After the complex solid model is segmented based on the feature framework and mapped onto individual hexahedral volume patches, these patches are stitched together to reconstruct a complex volumetric parametric model. When these hexahedral volume patches are stitched together, the differing parameter directions of the patches result in varying contact boundary faces. Consequently, the indices of the control points related to the boundary conditions differ across the patches, making the manual identification of relevant control points highly cumbersome. Therefore, an algorithm must be designed to automatically search for control points associated with boundary conditions.

**Fig. 8:**
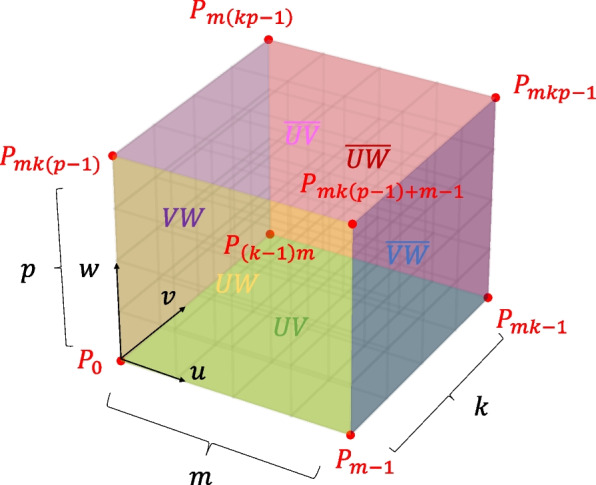
Six faces of a parametric volume

The volumetric model had three parametric directions: *u*, *v*, and *w*. Suppose that the number of control points in each direction are *m*, *k*, and *p*. During the connection, it is necessary to identify the type of common boundary surface, which falls into one of six categories: *UV*, *UW*, *VW*, and the opposite surfaces $\overline{UV}$, $\overline{UW}$, $\overline{VW}$. A hexahedral volume patch with six boundary surfaces and eight corner control points is illustrated in Fig. 8.

**Fig. 9:**
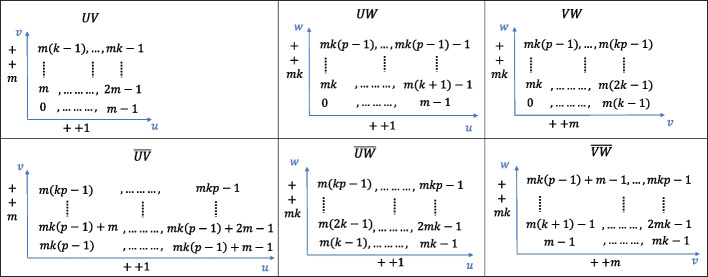
Control-point indices of six boundary surfaces

The control points are stored in a sequence starting from where the parameter is zero, arranged in the *u*-parameter direction. When the first row is filled, the process proceeds in the *v*-parameter direction. After completing the first layer, the arrangement continues upward along the *w*-parameter direction. The indices of the control points on different boundary surfaces are shown in Fig. 9.

After segmentation, the volumetric patches already satisfy the $G^0$ continuity. As shown in Fig. 4, the blue control points on the boundary surfaces of the two volumes have identical coordinates. First, it is necessary to search for the coinciding control points and store their indices. Based on these control-point indices, the type of contact boundary surface—whether *UV*, *VW*, or *UW* and their respective opposing surfaces—can be determined. As indicated by the previous Eqs. 5 and 7, to achieve *G*^1^ continuity, the rows of the control points adjacent to the boundary must satisfy certain conditions. Based on the indices of the common boundary control points and the types of contact surfaces, a row of internal yellow control points related to *G*^1^ continuity is generated by advancing within the volume in the direction perpendicular to the contact surface.

#### Merging algorithm of volume patches

**Fig. 10:**
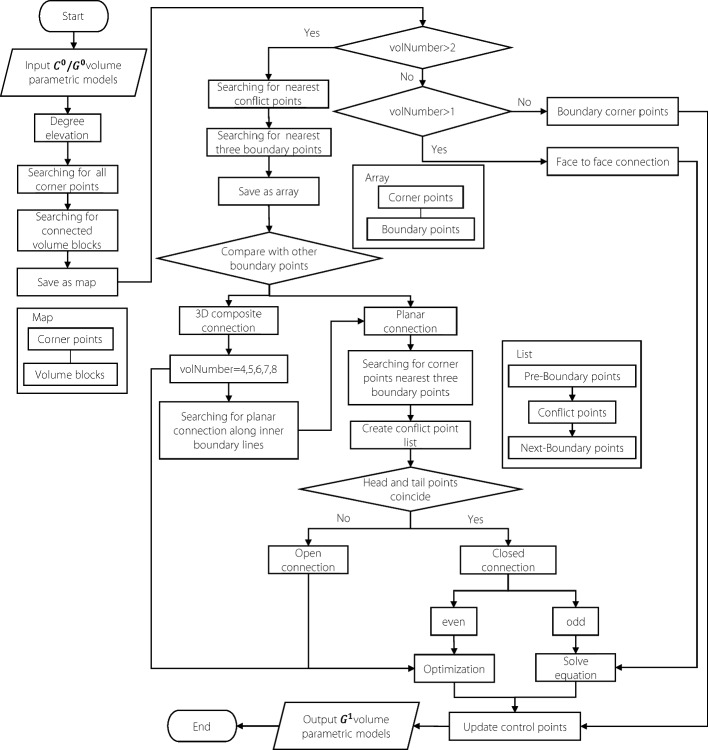
*G*^1^ continuity adjustment algorithm

This subsection presents an algorithm developed specifically to implement the *G*^1^ continuity connection method for volumetric parametric models as detailed in the preceding discussion. The algorithm was designed to automatically and accurately retrieve the positions of the boundary, corner, and conflict points based on the storage order of the control points in the *u*, *v*, *w* directions, and other information obtained from the volume parametric model. A flowchart of the algorithm is shown in Fig. 10.

**Algorithm steps**

Step 1: Input and initialization

1. Provide a volumetric parametric model with $C^0/G^0$ continuity.
2. Elevate the polynomial degree of the model to at least three to ensure that there are sufficient control points in each direction for adjusting continuity.

Step 2: Corner point detection and classification

3. Traverse and locate all corner points of the volume blocks.
4. Store each corner point and all blocks connected to it as a map structure.
5. Classify the connection type according to the number of connected blocks:
  1 block $\rightarrow$ Boundary corner point (no adjustment needed).
  2 blocks $\rightarrow$ Face-to-face connection (perform *G*^1^ adjustment).
  More than two blocks $\rightarrow$ Conflict control points exist (requires special handling).

Step 3: Identification of conflict points

6. For each conflict corner point, search for the corresponding conflict point — the internal control-point closest to the corner within adjacent blocks (Fig. 5).
7. For each conflict point, identify the three nearest boundary points of the volume block and store them as an array $\{P_c,P_{b1},P_{b2},P_{b3}\}$
8. By checking overlapping boundary points among different arrays, determine the connection type near the corner (planar multi-patch or 3D composite connection) as described in *G*^1^ continuity conditions of complex connections subsection.

Step 4: Handling of 3D composite connections

9. For 3D composite connections, address both the corner continuity and the planar connection along internal edges near the corner.
10. Select one boundary point as the rotation axis, and search for conflict points along the plane formed by the other two boundary points.
11. Construct a conflict point list in the form $\{P_{\text {pre}},P_{\text {conf}},P_{\text {next}}\}$.
12. Insert points into the list based on whether the rotation axes align:
  If the boundary point coincides with the head control-point, insert before the head node.
  Otherwise, insert after the tail node.
13. The resulting ordered conflict point list is used to identify whether the connection is closed (head and tail points coincide) or open (do not coincide).
14. Continue searching along internal boundary lines until all conflict points are detected.

Step 5: Continuity equation solving and optimization

15. As discussed in Planar multi-patches connection subsection, when an even number of volume patches are joined in a closed configuration or 3D composite connections, the coefficient matrix of the continuity equations becomes rank-deficient, leading to infinitely many solutions. To ensure a unique and stable solution, employ the Sequential Least Squares Programming optimization method to solve for the control points satisfying *G*^1^ continuity.
16. The optimization objective minimizes the deviation between the updated and original inner control points:
  8 $$\begin{aligned} \min \sum \limits _{i = 0}^{n} \left\Vert P_i^I-\tilde{P}_i^I \right\Vert ^2 \end{aligned}$$
  ensuring that the optimized model remains as close as possible to the original geometry.

Step 6: Output

17. Update the control points and regenerate the volumetric parametric model with *G*^1^ continuity.

## Results and Discussion

This section presents examples of applying the *G*^1^ continuity adjustment algorithm to volumetric parametric models. Currently, no unified standard exists for evaluating the quality of volumetric models. In this section, the volumetric models generated using reflection lines, Jacobian ratios, curvature maps, and isoparametric lines are evaluated.

**Fig. 11:**
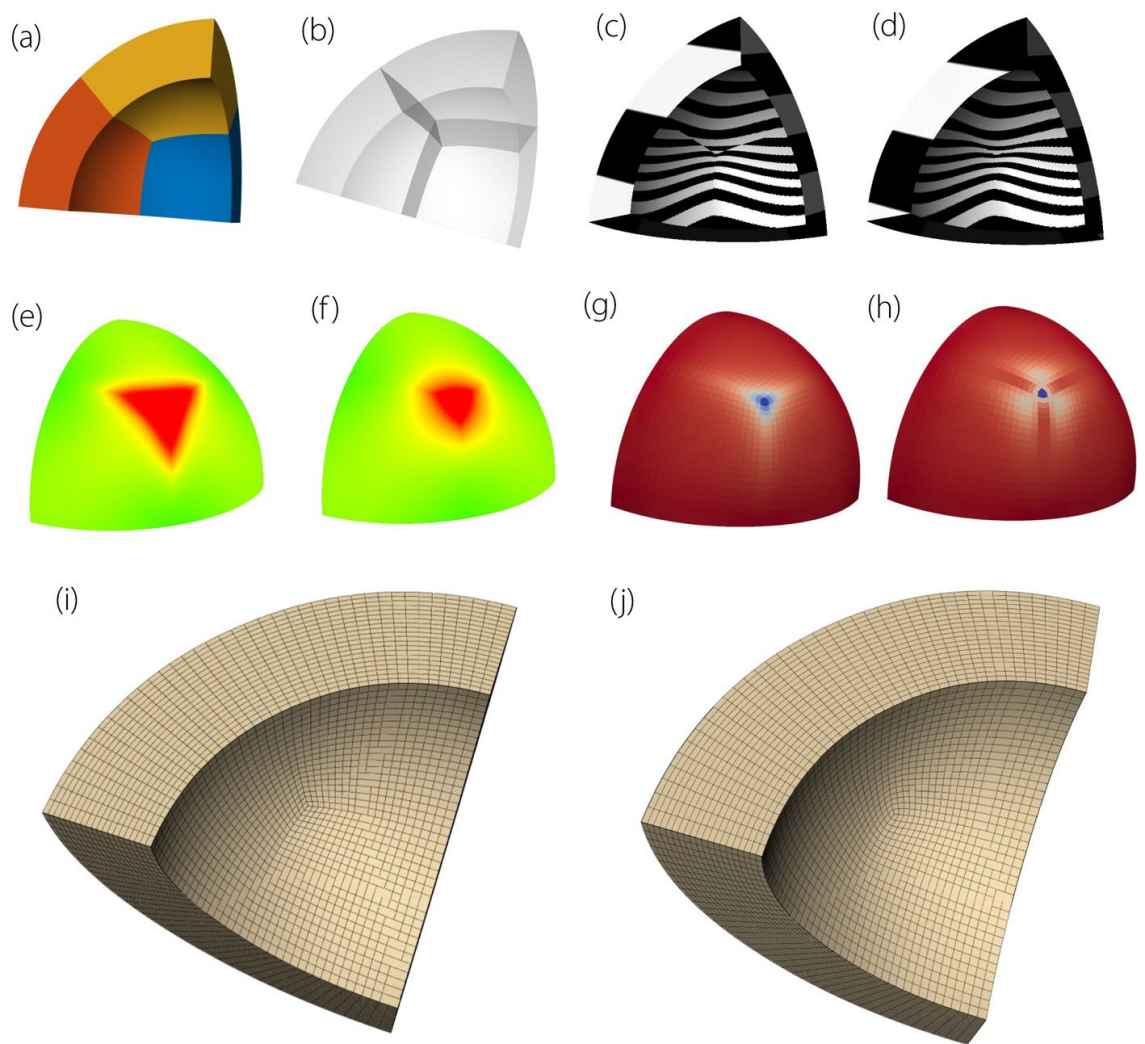
Three volume parametric patches. **a** original model, **b** transparent representation, **c** reflection line (before adjustment), **d** reflection line (after adjustment), **e** curvature map (before adjustment), **f** curvature map (after adjustment), **g** Jacobian ratio (before adjustment), **h** Jacobian ratio (after adjustment), **i** isoparametric lines (before adjustment), **j** isoparametric lines (after adjustment)

To facilitate the observation of changes before and after continuity adjustments, Fig. 11 shows the reflection lines, curvature distribution, Jacobian distribution, and isoparametric line representations of a simple three-patch volume parametric model. This setup corresponds to a closed planar connection case with an odd number of patches, as discussed in Planar multi-patches connection subsection, allowing the control points to be solved directly from the equations. The comparison shows that, in the original model, the inflection and isoparametric lines are discontinuous at the connections. After adjustment, they become continuous. In addition, the curvature distribution is more uniform, and the range of negative Jacobian values is reduced, reflecting an improvement in model continuity.

The proposed method could also be applied to complex volumetric parametric models. Continuity adjustments were performed on complex models containing both open and closed planar connections, and 3D composite connections generated through both the creation and recreation methods. Table 1 provides a detailed summary of the numbers of blocks and connection types, and the generation times for each model. It can be observed that models with fewer blocks are generated more quickly, whereas those with a larger number of blocks require longer generation times, which do not meet the requirements for interactive operations. Therefore, further improvements in the computational efficiency are necessary in the future, for example, by employing GPU acceleration or other optimization techniques.

**Table 1:** Number of patches and connection type of volume parametric models

Construction method	Name	Patch	3D composite connection	Planar connection	Generation time
Creation method	Bearing seat	26	51	54	0.07 s
	Automobile part	192	304	296	0.118 s
	Vane Wheel	24	36	36	0.038 s
	Reducer	440	727	458	6.576 s
Recreation method	Rod	56	114	122	1.784 s
	Deckle	16	34	46	0.861 s
	Cup	80	114	118	0.053 s
	Joint	64	106	113	0.252 s

**Fig. 12:**
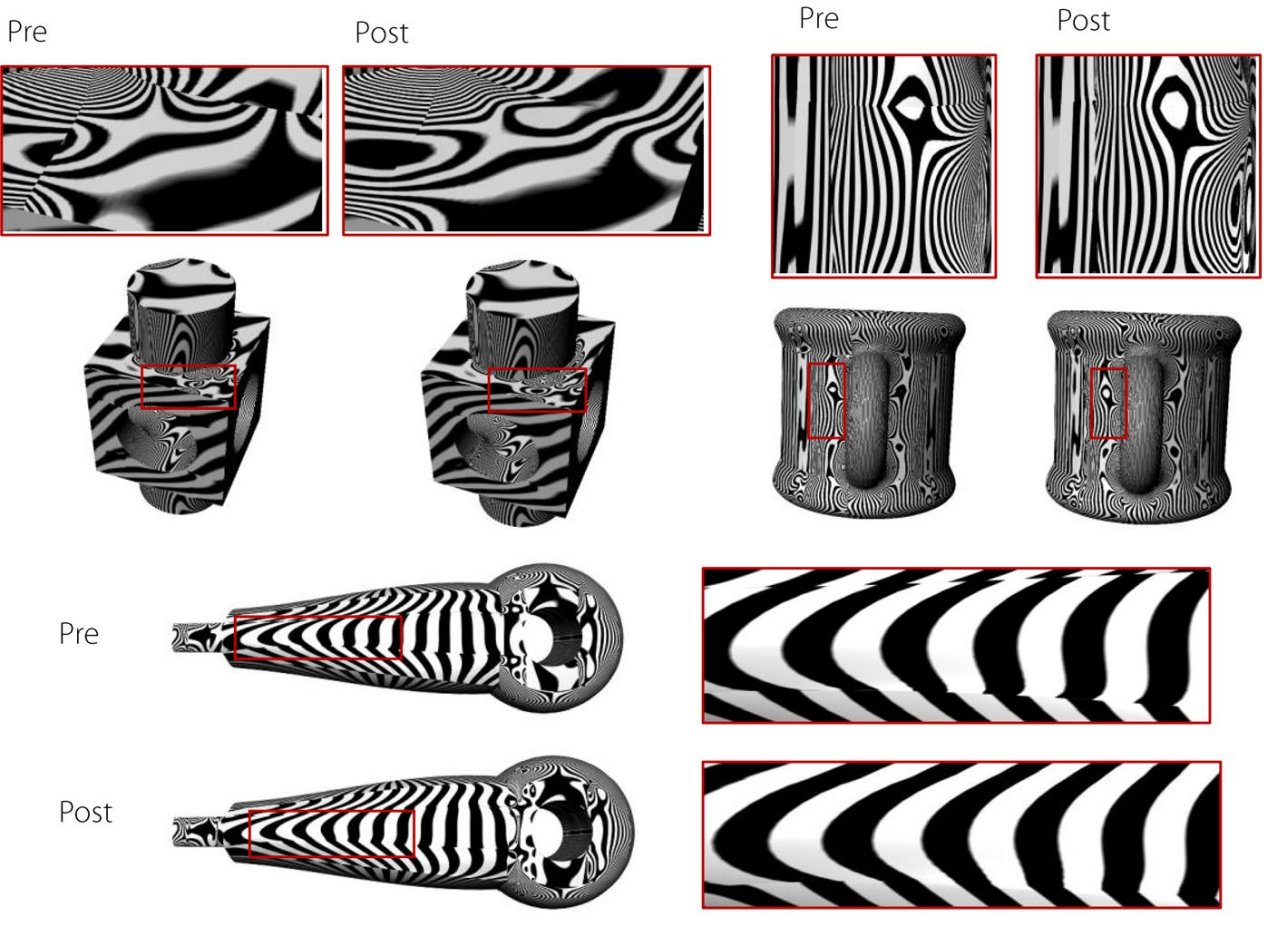
Evaluating the quality of volumetric parametric models with reflection lines

**Fig. 13:**
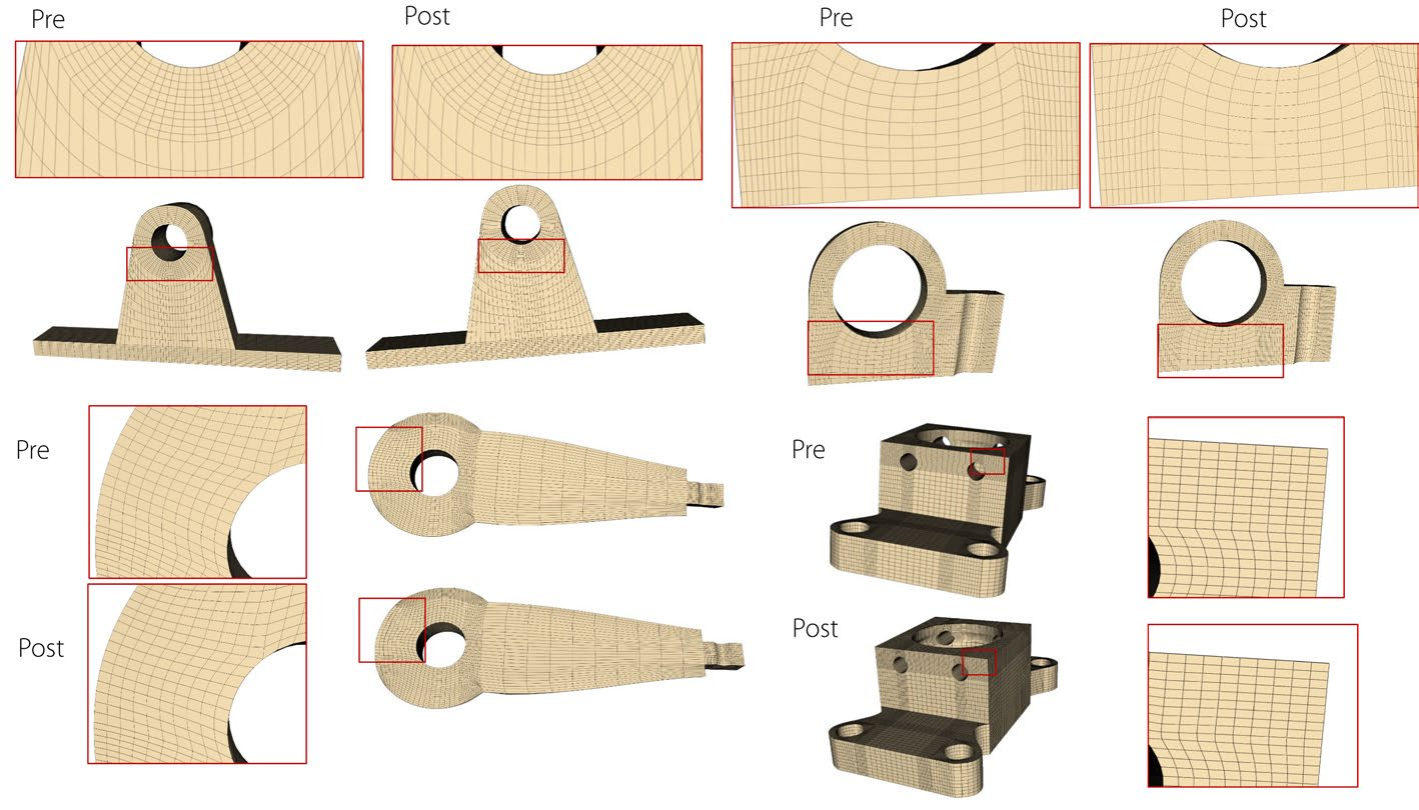
Evaluating the quality of volumetric parametric models with isoparametric lines

Figures 12 and 13 compare the reflection and isoparametric lines before and after applying a continuity-adjustment algorithm to a complex volumetric parametric model.

These examples demonstrate that the proposed method can enhance the continuity of volumetric parametric models with complex multidirectional connections generated through both the creation and recreation methods. Additionally, the generated volumetric models are based on NURBS, allowing the extracted model boundaries to be displayed in mainstream CAD software.

**Fig. 14:**
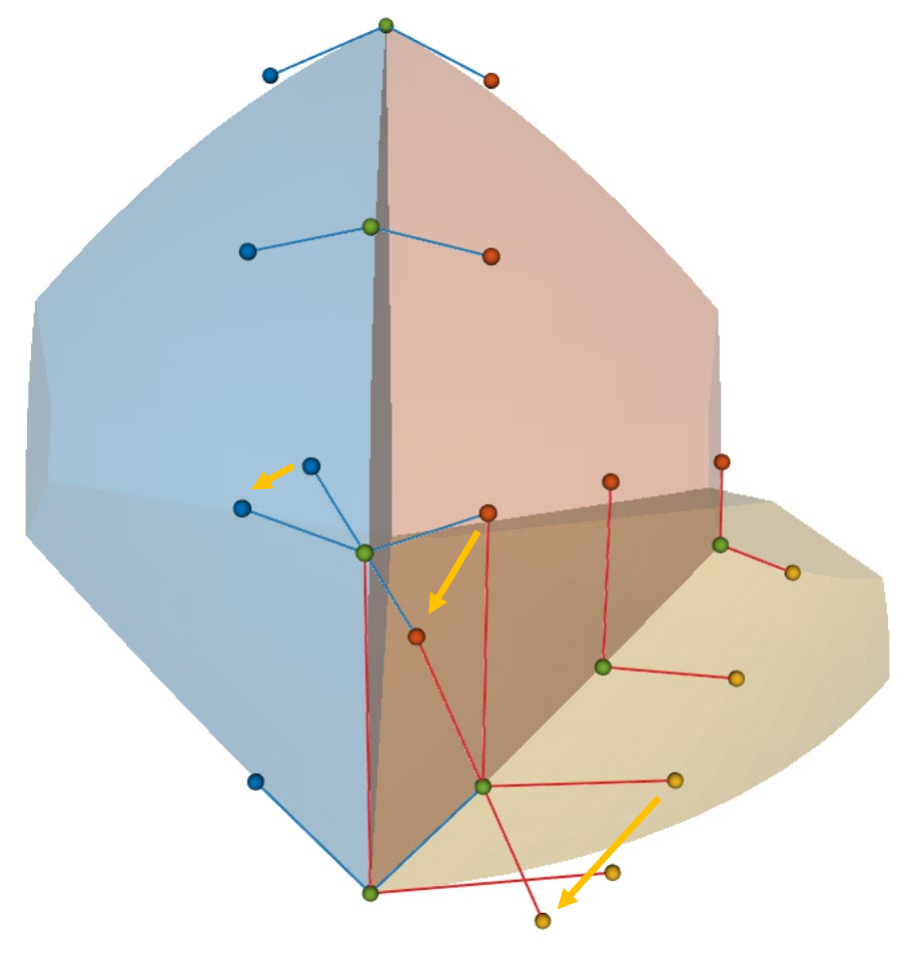
Non-adjustable volume parametric model

However, the proposed method has certain limitations. For example, there are specific constraints on the models that can be adjusted. The aspect ratio of the volumetric blocks should not be excessively large and should ideally approximate a regular hexahedron. Additionally, the angle formed between the boundary control points of adjacent blocks and the neighboring internal control points should not be too wide. As shown in Fig. 14, the three-patch volume parametric model has an excessive aspect ratio, approaching a trapezoidal shape. This results in a large angle between the lines connecting the control points, making it difficult for the conflict control points in the middle patch to simultaneously satisfy the continuity conditions of both boundary surfaces. The hexahedral blocks of the volumetric parametric models generated using the segmentation-mapping-merging mechanism and simplified polycube method have a more regular shape, rendering them suitable for the proposed continuous adjustment algorithm.

## Conclusions

This study proposes a systematic method for constructing complex volumetric parametric models and achieving *G*^1^ continuity through control-point adjustments. This approach provides an effective solution for enhancing the quality of models generated by both the creation and recreation methods and can accommodate various complex connections present in volume parametric models. The resulting examples demonstrate significant improvements in model quality, rendering them well-suited for analysis and production requirements. Future work will focus on further enhancing the continuity of volumetric parametric models to $G^2$, and employing additional optimization methods to eliminate negative Jacobian values.

## Data Availability

Data will be made available on request.

## Abbreviations

CAD: Computer aided design
CAE: Computer aided engineering
IGA: Isogeometric analysis
B-rep: Boundary representation
V-rep: Volumetric representation
STEP: Standard for the exchange of product model data
AS *G*^1^: Analysis-suitable *G*^1^
iMSO: Integrated design software for modeling, simulation and optimization
GB: Generalized Bézier
